# Age-related changes to brain energetics revealed by PET/MRI imaging

**DOI:** 10.1016/j.nicl.2026.104014

**Published:** 2026-05-27

**Authors:** Connor W.J. Bevington, Sahib Dhaliwal, Jessamyn McKenzie, A. Jon Stoessl, Vesna Sossi

**Affiliations:** a Department of Physics and Astronomy, University of British Columbia, 325-6224 Agricultural Road, Vancouver, BC V6T 1Z1, Canada; b Pacific Parkinson’s Research Centre, University of British Columbia, Vancouver, BC, Canada; c Department of Medicine, Division of Neurology, University of British Columbia, Vancouver, BC, Canada; d Djavad Mowafaghian Centre for Brain Health, University of British Columbia, Vancouver, BC, Canada

**Keywords:** Brain energetics, Aerobic glycolysis, PET/MRI, Healthy aging, Neurodegeneration, Spatial covariance analysis

## Abstract

Aging is accompanied by several neurophysiological changes. Of recent interest are age-related changes in brain energetics—how the brain produces and uses energy. In vitro, preclinical, and genetic investigations point to a general decline in the efficiency of brain energetics in aging, which may help explain age-related changes in mood, cognition, movement, and behaviour, as well as an increased likelihood of developing neurodegenerative disorders. We hypothesized that age-related alterations to brain energetics are observable using functional neuroimaging data from healthy individuals (N = 24, 35–80 years), and that alterations may occur in regions associated with neurodegenerative pathology. Specifically, we jointly analyzed cerebral glucose metabolism and blood flow data, defining novel metrics that capture regional variability in processes related to relative energy production (rEP) and relative aerobic glycolysis (rAG), an energy production mechanism. By applying Scaled Subprofile Modeling Principal Component Analysis (SSM-PCA) to these metrics, we identified spatial covariance patterns that capture a widespread age-related restructuring of brain energetics. Broadly, an age-related rEP pattern (r_age_^2^ = 0.72) consisted of rEP increases in frontal, basal ganglia, and brainstem regions, coupled with decreases in occipitoparietal regions, while an age-related rAG pattern (r_age_^2^ = 0.65) revealed rAG increases in substantia nigra and occipitoparietal regions and decreases in caudate and frontal regions. Finally, by comparing these patterns to well-known metabolic patterns related to Parkinson’s and Alzheimer’s disease, we identified the strongest topological similarity and covarying pattern expression between the age-related rEP pattern and the Parkinson’s Disease Related Pattern (PDRP). These results provide in vivo support for altered brain energetics with age and potential neurophysiological similarities between aging and neurodegeneration.

## Highlights

• We introduce novel metrics of relative energy production (rEP) and relative aerobic production (rAG).
• Spatial covariance pattern analysis captures age-related alterations in these measures.
• A subset of these regional changes show similarity to well-known neurodegenerative alterations.

## Graphical abstract

**Figure:**
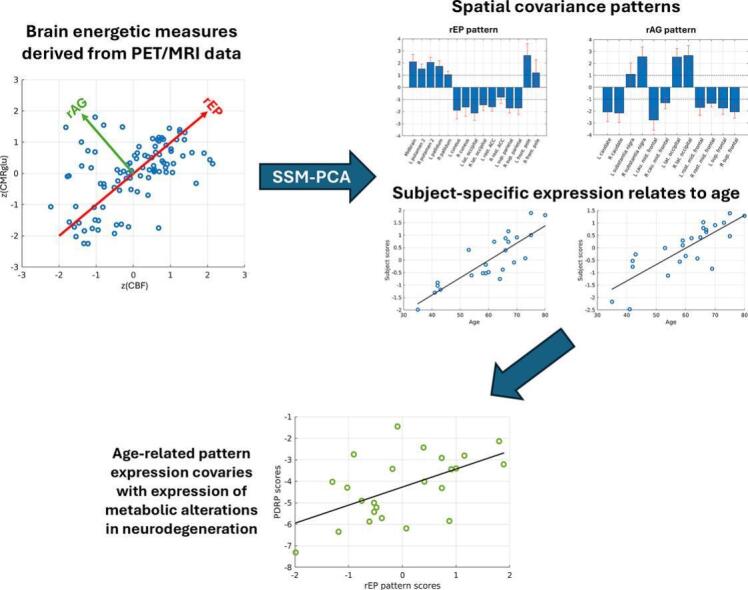

## 1 Introduction

### 1.1 The aging brain

We currently live in a rapidly aging society. However, this increased longevity comes with a general decline of brain and body health as well as increased incidence of age-related disorders (Hou et al., 2019, Sugimoto et al., 2022, Gielen et al., 2023), thus requiring an urgent and coordinated public health response (World Health Organization 2015). Since the brain helps control processes related to mood, cognition, movement, and behaviour, understanding the underlying neurophysiological changes associated with aging may help guide this response in several ways: (i) biomarkers of age-related brain changes may lead to a better classification and earlier identification of healthy vs. unhealthy aging, as has been recently observed with structural imaging (Bethlehem et al., 2022); (ii) identifying brain-related aging mechanisms may aid in the development of targeted therapeutic interventions to slow aging; and (iii) brain alterations associated with aging can be compared to those involved in neurodegenerative disorders, to infer possible commonalities between aging and disease and thus gain possible insights into age-related disease risk factors.

One aspect of brain function with increasing in vitro, preclinical, and genetic evidence of alteration with age is brain energetics—the production and utilization of energy in the brain (Cui et al., 2012, Camandola and Mattson, 2017). This may be unsurprising: the brain has a relatively high energy demand required to coordinate and regulate multiple processes, so if age alters the energy production mechanisms that maintain normal brain function, this may be observable through a degradation of these processes.

### 1.2 A brief primer on brain energetics

Energy for neuronal function is provided by adenosine triphosphate molecules (ATP), which are produced by metabolizing glucose molecules, either in the presence (aerobic energy production) or absence of oxygen (anaerobic energy production). Oxidative phosphorylation is the aerobic energy production mechanism, which produces approximately 32–34 ATP for each glucose molecule metabolized. By comparison, glycolysis—the anaerobic energy production mechanism—produces only two ATP per glucose molecule. Because of high neuronal energy demand, oxidative phosphorylation is thus preferred over glycolysis for its higher ATP output (Allaman and Magistretti, 2012, Magistretti and Allaman, 2015).

Despite this preference, there is in vivo evidence that the brain will still resort to glycolysis *despite* the presence of adequate oxygen (Vaishnavi et al., 2010, Goyal et al., 2017), a mechanism known as aerobic glycolysis (AG) (Warburg 1956). Several physiological reasons have been proposed for these observations. First, oxidative phosphorylation produces radical oxygen species which, in high amounts, can lead to oxidative stress and ultimately neuronal damage (Allaman and Magistretti, 2012). As a result, AG may occur in highly energetic neurons as a protective mechanism to limit oxidative stress (Brand and Hermfisse 1997). Additionally, since brain processes are spatiotemporally dynamic in nature, the *rate* of energy production across the brain and time varies dramatically. Though glycolysis produces less ATP in absolute terms, it produces ATP *faster* than oxidative phosphorylation, meaning AG may be upregulated for rapid energetic needs of the brain, such as during task performance (Fox et al., 1988, Madsen et al., 1995, Vlassenko et al., 2006). Finally, AG is proposed to be involved in synaptic growth and remodeling, which is supported by AG increasing during childhood and, once in adulthood, being elevated in regions that retain gene expression typical of infancy (i.e., transcriptional neoteny) (Goyal et al., 2014).

### 1.3 Brain energetics and aging

Aerobic energy production occurs in the mitochondria of neurons; given the increasing genetic and in vitro evidence for mitochondrial dysfunction with age (Cui et al., 2012, Jang et al., 2018, Miwa et al., 2022, Guo et al., 2023), it is reasonable to hypothesize that energy production mechanisms change with age, which would lead to changes in the preference for AG and thus potentially the magnitude of energy produced. Recent ex vivo maps have demonstrated large variability in mitochondrial density across brain regions (Mosharov et al., 2025), which could lead to regional variability in these changes as well. Furthermore, links between mitochondrial dysfunction and neurodegenerative disorders have also been proposed and/or identified (Powers et al., 2007, Carmo et al., 2018,, Ashleigh et al., 2023, Alqahtani et al., 2023, Klemmensen et al., 2024), and age tends to be an important risk factor for developing these diseases (Hou et al., 2019). Associations between aging, neurodegeneration, and mitochondrial dysfunction suggest age-related changes to AG and overall energy production may preferentially occur in regions susceptible to different forms of neurodegeneration, potentially contributing to the development and progression of pathology.

Spatial quantification of brain energetics can be explored in vivo by computing parametric images of metabolic rate of glucose (CMRglu) and metabolic rate of oxygen (CMRO2) from neuroimaging data. Based on the stoichiometry of cellular respiration, the oxygen-to-glucose ratio (i.e., CMRO2/CMRglu) would be six if glucose is fully oxidized, and thus a value less than six indicates some degree of AG. The magnitude of CMRglu and CMRO2 can also offer insights into the overall energy production. CMRO2 is a function of cerebral blood flow (CBF) and the oxygen extraction fraction (OEF). The spatial distribution of OEF is essentially uniform in the healthy brain (Gauthier and Hoge, 2012, Wise et al., 2013, Cho et al., 2021, Fan et al., 2020); regional variability in CMRO2 is thus driven by regional variability in CBF.

Previous investigations have demonstrated that CMRglu, CBF, and CMRO2 all tend to decline with age (Aanerud et al., 2012, Mosconi, 2013, Goyal et al., 2017, Seifer et al., 2018; Mokhber et al., 2021; Weistuch et al., 2021, Wu et al., 2024). Regional differences in the rate of decline likely exist; this has been captured by pattern analysis investigations which characterize the topology of age-related changes after global effects have been removed. Specifically, age-related CMRglu patterns revealed frontal metabolism declines faster than in occipito-parieto-temporal regions (Moeller et al., 1996, Matthews et al., 2018) and age-related CBF patterns showed stronger declines in frontal, parietal, and cerebellar regions relative to occipital and temporal regions (Zhang et al., 2018). However, there has been limited direct investigation of the spatial concordance of these changes, which is important for the investigation of (potentially) spatially variant age-related changes to energy production mechanisms, rather than observing the individual metabolic processes contributing to brain energetics in isolation. Direct voxelwise or regional division of CMRO2 by CMRglu would, in theory, provide a direct insight into brain energetics—particularly AG. However, in practice this is impractical due to the noise in acquired data and modeling assumptions used to generate these parametric images, which leads to limitations in their quantitative accuracy and precision.

A previous PET study circumvented these challenges by normalizing CMRglu and CMRO2 to global utilization rate—i.e., focusing on *spatial distributions* of these metrics rather than absolute values—then obtaining the residuals after regression of these normalized metrics (Vaishnavi et al., 2010). The results of that study identified a spatially varying pattern of AG in the healthy human brain, characterized by relatively elevated AG in frontal, parietal, and lateral temporal regions, coupled with relatively lower AG in cerebellum and inferior temporal gyrus. However, the cohort studied only included younger adults and thus it is unclear whether this pattern of AG is retained with aging. Another study suggested that global AG decreases with age and the largest changes occur in regions with the highest AG in youth, but the methodology was limited by scaling regional data with age-dependent literature averages of CMRglu and CMRO2 (Goyal et al., 2017).

The work reported here aims to combine the benefits of the two approaches above, namely by (i) acquiring multiple imaging measures related to brain energetics in subjects without resorting to literature averages, and (ii) identifying patterns of age-related changes in brain energetics by acquiring these data in individuals over a wide age range. Given that age is an important risk factor for neurodegenerative disorders, this work further aims to investigate whether these changes in age-related brain energetics relate to established metabolic patterns of neurodegenerative disease described in the literature—specifically the Parkinson’s Disease Related Pattern (PDRP) (Ma et al., 2007), the Parkinson’s Disease Cognitive Pattern (PDCP) (Huang et al., 2007), and the Alzheimer’s Disease Related Pattern (ADRP) (Teune et al., 2014)—as Parkinson’s and Alzheimer’s disease are the fastest growing (Dorsey et al., 2018) and most prevalent (Liu et al., 2025) neurodegenerative disorders worldwide, respectively, and both exhibit rapid increases in incidence with age (Hou et al., 2019). Additionally, the expression of PDRP-like patterns has also been shown to correlate with age—within both diseased and healthy control groups (Bevington et al., 2026)—and spatial overlap is observed between age- and PD-related metabolic patterns (Moeller et al., 1996, Matthews et al., 2018). Therefore, it is a reasonable hypothesis that aging effects, such as changes in brain energetics, may contribute towards a continuum between “normal” metabolism and metabolism affected by disease (as characterized by disease-related patterns). Therefore, it is relevant to compare the metabolic topography of disease with the topography of age-related changes in brain energetics.

To achieve this, we introduce two novel measures sensitive to regional variability in energy production and AG, derived from CMRglu and CBF data acquired in a single PET/MRI scanning session. An initial qualitative analysis is performed to identify (i) regional trends in energy production and AG across the major subdivisions of the brain and (ii) age-related directional shifts in energetics by comparing these measures in older vs. younger subjects. For a more quantitative analysis of aging effects, we then enter these measures into a pattern analysis framework which is designed to identify spatial patterns whose expression maximally separates older vs. younger subjects, thus capturing the set of regional changes in brain energetics most related to aging. We then discuss the implications of these regional changes in the context of aging, and finally any similarities to PDRP, PDCP, and/or ADRP are highlighted to identify shared pathology between neurodegeneration and aging.

## 2 Methods

### 2.1 Subject recruitment and demographics

24 healthy individuals were recruited as controls in a separate study investigating brain energetics in Parkinson’s disease, approved by the UBC Clinical Ethics Review Board under Application No. H19-01839, following the ethical principles of the Declaration of Helsinki. Written informed consent was obtained prior to enrollment in the study. Subjects had a broad age range (35–80 years; mean: 60.2 years; standard deviation: 12.2 years) permitting an investigation of age-related alterations. Subject exclusion criteria included any chronic diseases, an immediate family history of neurodegenerative disorders, or inability to be scanned in a 3T MRI environment.

### 2.2 Data acquisition and processing

Subjects underwent a 60-minute, 5 mCi [^18^F]fluorodeoxyglucose (FDG) scan on the GE SIGNA PET/MR in the eyes open condition. Simultaneous MRI data were acquired; relevant to this study were a T1-weighted MPRAGE sequence (TR/TE/TI = 2700/3.12/1000 ms, flip angle 8°, 1 mm isotropic voxels, 202 slices) to provide an anatomical reference and the vendor’s standard pulsed continuous Arterial Spin Labelling sequence (GE 3D ASL: TR = 4965 ms, TE = 11 ms, 1450 ms labeling time, 1525 ms post-label delay, flip angle 111.1°, bottom-up acquisition, 1.72 mm x 1.72 mm in-plane voxel size, 3.5 mm slice thickness, 46 slices) which results in vendor-estimated CBF images.

Dynamic FDG images were obtained by grouping the FDG data into 4 x 1 min, 3 x 2 min, 8 x 5 min, 1 x 10 min time frames and reconstructing using the denoising reconstruction PSF-HYPR4D-K-TOFOSEM (Cheng et al., 2022) with 10 iterations, 28 subsets, and 1.39 mm x 1.39 mm x 2.78 mm voxel size. Rigid frame-to-frame realignment was performed using SPM12 to account for any motion during the scan. Voxelwise maps of CMRglu were computed as:

(1) $$\mathrm{CM}R_{\mathrm{glu}}=K_{i}\times \frac{C_{\mathrm{glu}}}{\mathrm{LC}}$$

where $K_{i}$ is the net FDG uptake rate constant obtained using the Patlak method (Patlak et al., 1983) with an image-derived plasma input function (Cheng et al., 2022), LC=0.65 is the “lumped constant” accounting for differences in phosphorylation between glucose and FDG, and $C_{\mathrm{glu}}$ is the plasma glucose concentration obtained from blood samples. As detailed in Cheng et al. (2022), a spatiotemporal constraint is used to select voxels for the image-derived input function. Initially, voxels are selected by thresholding a maximum intensity projection of image slices spanning the carotid artery, then a subset of these voxels are retained such that the corresponding time activity curve maximizes the agreement with three venous blood samples taken approximately 30, 45, and 60 min post-injection (where arterial-venous differences are minimal).

The CMRglu and CBF images were co-registered to each subject’s T1-weighted image with affine transforms with six degrees of freedom using FSL’s “FLIRT” function. Freesurfer was used to segment subject-specific T1 images into cortical and subcortical regions of interest (ROI) (Fischl, 2012) for CMRglu and CBF regional quantification. All analyses were conducted in subject space to minimize interpolation errors and segmentation difficulties that could arise from using a common standard template across a wide age range (Richards et al., 2016). Considering our hypothesis that changes in age-related energetics may occur in regions involved in neurodegeneration, we manually subdivided the putamenal ROIs into three subregions along the anterior–posterior axis, as gradients in pathology have been observed along this axis in neurodegeneration and aging (Oh et al., 2012, Drori et al., 2022, Wen et al., 2025). Specifically, this subdivision was performed by applying k-means clustering with three clusters to the distribution of anterior-posterior voxel indices of the Freesurfer putamen segmentation, using the “kmeans” function in MATLAB. Relatedly, the spatially broad Freesurfer brainstem ROI was replaced by separate ROIs for the pons, medulla, midbrain, and bilateral substantia nigra (SN). These ROIs were hand-drawn by an expert neurologist on a reference T1 image. A subject-specific warping between the T1 image of each subject and this reference T1 image was computed using the “flirt” and “fnirt” functions in FSL, then the inverse warping was applied to map the reference brainstem ROIs to subject space. All automated and modified ROIs were visually inspected to ensure reasonable positioning when overlaid on the T1, CMRglu, and CBF images. These modifications resulted in a total of 97 ROIs for analysis, which are listed in the Supplementary Information.

### 2.3 Metrics relevant to brain energetics: Analyzing concurrence and discordance of CMRglu and CBF

**Fig. 1:**
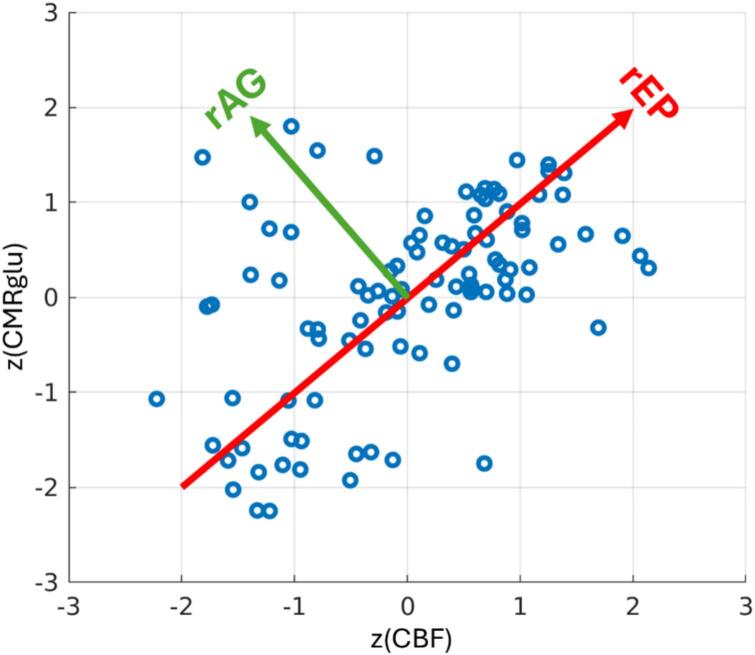
Derivation of rEP and rAG for a sample subject. Each point corresponds to the z-score normalized CMRglu and CBF for each region.

Following (Shokri-Kojori et al., 2019), we performed a simple data rotation to analyze the concurrence and discordance of the spatial distributions of CMRglu and CBF (visualized in Fig. 1). Mathematically, rEP and rAG are computed as:

(2) $$\left[\begin{array}{c} \mathrm{rEP}\\ rAG \end{array}\right]=\left[\begin{array}{cc} \cos45^{{}^{\circ}} & \sin45^{{}^{\circ}}\\ -\sin45^{{}^{\circ}} & \cos45^{{}^{\circ}} \end{array}\right]\left[\begin{array}{c} z(CBF)\\ z(CMR_{\mathrm{glu}}) \end{array}\right]$$

where z(·) indicates the z-score, taken over all regions. As seen in Fig. 1, in the space of [z(CBF), z(CMRglu)] this 45° rotation amounts to a change of basis where rEP is the projection of the normalized data onto the line of unity (thus capturing concurrence) whereas rAG is an orthogonal measure of the distance away from unity (thus capturing discordance). Therefore, the regional values of CMRglu and CBF are transformed to a region-specific value of concurrence and discordance. Regions that concurrently have high (low) CMRglu and CBF relative to their brain averages indicate a high (low) degree of energy production. We thus denote the concurrence metric as relative energy production (rEP). Discordance between CMRglu and CBF instead offers insights into energy production mechanisms. A region where relative CMRglu exceeds relative CBF may be indicative of a relative preference for glycolysis; as CBF will in general be tightly linked to oxygen utilization, under the assumption of spatially uniform OEF this may further suggest a region of relatively elevated aerobic glycolysis. We thus denote the discordance metric as relative aerobic glycolysis (rAG).

### 2.4 Exploring regional and age-related trends in rEP and rAG

We separated our subjects into a younger (<=60 years; n = 11; 49.6 +/- 9.1 years) and an older (>60 years; n = 13; 69.2 +/- 5.2 years) cohort and separately averaged regional rEP and rAG within each group. The 60 years cut-off value between cohorts was chosen to maximize the difference in mean age between the cohorts while keeping subject numbers in the two groups approximately equal. We also grouped the Freesurfer ROIs into brain stem, cerebellum, frontal, temporal, parietal, occipital, and limbic groups to visualize potential regional clustering of rEP and rAG. As an initial investigation into effects of aging, we subtracted the averaged data of the younger group from those of the older group to observe the directional shift of rEP and rAG with aging.

### 2.5 Identifying age-related patterns of rEP and rAG

For a more robust and targeted analysis of age-related effects, Scaled Subprofile Modeling Principal Component Analysis (SSM-PCA) (Moeller and Strother 1991) was used to capture age-related variance in regional rEP and rAG. SSM generates a residual profile by demeaning the data across subjects and regions.^1^ This removes effects common across subjects and instead focuses on cohort differences; since all subjects were deemed healthy, a large source of variability is expected to be age-related. PCA then decomposes the residual profile into a set of spatial patterns (weights for each region, describing a relative increase or decrease of the metric) and subject scores (quantifying the expression of each pattern for each subject).

The spatial patterns are rank-ordered by the variance accounted for (VAF) in the dataset, with spatial patterns being linearly independent. This property of PCA means that not all age-related variance will necessarily be confined to a single pattern. One can assess the relevance of age in each pattern through the correlation between the associated subject scores and subject ages. However, to cohesively capture age-related changes in rEP and rAG in a single spatial pattern, we recombined the PCA components in a logistic regression model whose objective is to find a combined pattern with subject scores that maximally separate the younger and older subjects. Following Spetsieris and Eidelberg (Spetsieris and Eidelberg 2011), PCA components were only considered for the logistic regression model if they (i) explain > 5% VAF and (ii) separate younger vs. older via their subject scores with p < 0.2 (two-tailed Student’s *t*-test), to avoid spurious combinations. PCA components were combined in a stepwise fashion using Akaike Information Criteria (AIC) to determine the optimal combination (i.e., a component is retained in the model if it lowers AIC). Furthermore, 500 iterations of five-fold cross validation were used to obtain a robust pattern, which was performed as follows: (i) for each iteration, subjects were randomly sorted into five equal subsets; (ii) four out of five subsets were used to identify a combined spatial pattern as described above; (iii) a final spatial pattern was obtained by computing the mean and standard deviation across the spatial pattern weights from each iteration; (iv) final subject scores were obtained by projecting this final pattern onto the SSM-processed data of all subjects. This approach minimizes the likelihood of overfitting by pre-filtering PCA components before consideration in the logistic regression model and the use of AIC for determining the optimal component combinations for each cross-validation iteration. The cross-validation procedure itself intrinsically provides an analysis of the importance and stability of the spatial weights in the final pattern; a region with consistently high (or low) weighting across cross-validation iterations can be viewed as a salient signal contributing to an age-related topographic profile.

Covariance patterns can also be derived directly from correlations between regional rEP and rAG data and relevant clinical variables (i.e., age and other variables potentially associated with brain energetics, such as biological sex), using multivariate methods such as Partial Least Squares Correlation analysis (PLSC). While chronological age is inherently continuous, individual aging trajectories may vary, which manifests as additional variance when analyzing correlations between aging profiles and chronological age. The SSM-PCA approach described above does not explicitly enforce a correlation with chronological age and instead seeks a spatial pattern that separates older vs. younger subjects at the group level. A potential disadvantage of this approach is the potential to capture covariance unrelated to aging that nevertheless separates the two groups. Therefore, as a supplementary analysis we performed PLSC separately on rEP and rAG using age and biological sex as clinical variables. Broadly, PLSC applies Singular Value Decomposition to the correlation matrix between regional rEP/rAG and the clinical variables, resulting in paired spatial and clinical patterns whose subject scores covary. Specific mathematical details and cross-validation procedures can be found in the Supplementary Information and are based on the methodology presented in (Krishnan et al., 2011). Analysis code for computing rEP/rAG and running the SSM-PCA and PLSC analyses were developed in house using MATLAB and will be made available upon request.

### 2.6 Comparison of derived age-related patterns with neurodegenerative disease-related patterns

Spatial similarity between the derived rEP/rAG patterns and neurodegenerative disease-related patterns (PDRP, PDCP, and ADRP) was examined by correlating the spatial weights between the age-related patterns and those of each disease-related pattern using Spearman’s ρ, since topological similarities may not follow a linear relationship.

Similarly, we tested the hypothesis that the expression of age-related and disease-related patterns covary, such that a subject with higher expression of an age-related pattern would also tend to have higher expression of a disease-related pattern, and vice versa. This was characterized by exploring correlations between rEP/rAG subject scores and those from the disease-related patterns using Pearson’s ρ. Subject scores for the disease-related patterns were obtained using the Topographic Profile Rating technique (Spetsieris et al., 2006), adapted for our regional data. This proceeded as follows: for each of PDRP, PDCP, and ADRP, the voxelwise disease-related pattern and group mean profile—both defined in standard space—were averaged over the same set of Freesurfer ROIs described in Section 2.2, to yield a regional disease-related pattern (rDRP) and group mean profile (rGMP). For each subject, regional CMRglu data was log-transformed and then regional global mean rate (rGMR) was computed by averaging across the regional log-transformed data [log(rCMRglu)]. Regional subject residual profiles (rSRP) were computed as:

(3) $$rSRP=\log\left(rCMRglu\right)-rGMR-rGMP$$

which were subsequently projected onto the rDRP to provide prospective subject scores. Note that we are only interested in potential covariance between these prospective subject scores and the rEP/rAG subject scores, so no standardization was applied to the prospective subject scores (i.e., their magnitude is not clinically meaningful).

This latter comparison assesses whether the expression of age-related brain energetics alterations helps explain the variability in disease-related pattern expression; though these subjects are healthy controls and thus are expected to have relatively low expression of disease-related patterns, there is still considerable variability in their individual disease-related pattern expression.

## 3 Results

### 3.1 Regional trends in rEP and rAG

**Fig. 2:**
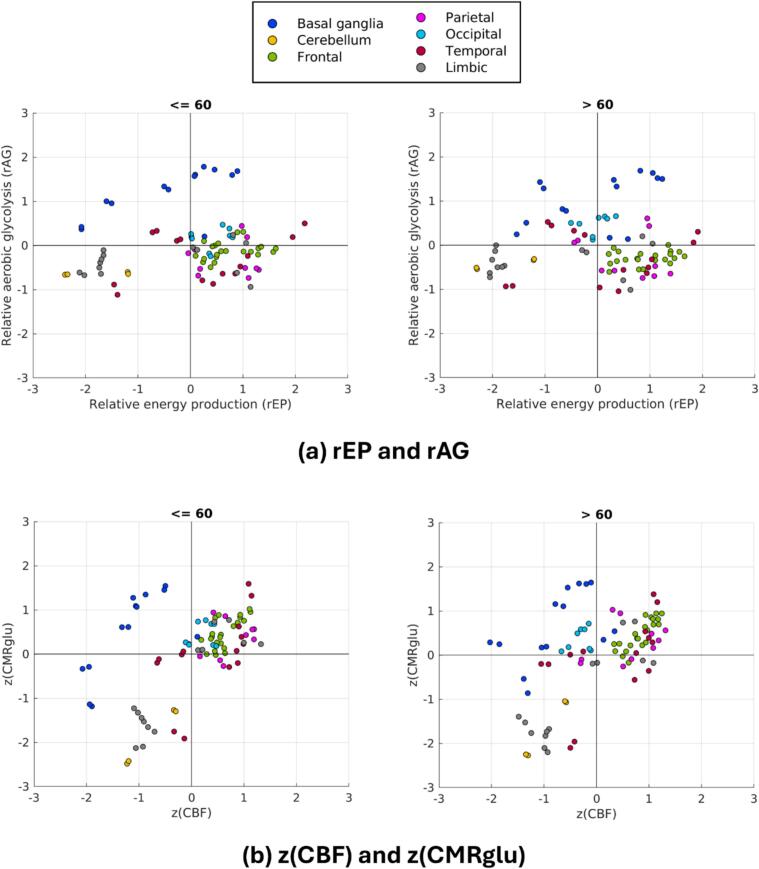
Clustering of regional brain energetics parameters for younger (<=60) and older (>60) subjects in the space of **(a)** [rEP, rAG] and **(b)** [z(CMRglu), z(CBF)].

Fig. 2a reveals regional clustering of rEP and rAG for both the younger and older cohort within the structural subdivisions of the brain. Fig. 2b reproduces these data in the space of z(CMRglu) and z(CBF) to illustrate how these relate to the observed trends in rEP and rAG. A complete list of regional z(CMRglu), z(CBF), rEP, and rAG is provided in the Supplementary Information. Focusing on the older group, the regional clustering is summarized in Table 1. Briefly, rEP is highest in regions of the default mode network and anterior putamen, and lowest in cerebral white matter, cerebellum, globus pallidus, and SN. rAG is highest in basal ganglia, where CMRglu is generally above brain average but CBF is significantly below brain average, and lowest in cerebellum, where for grey matter z(CMRglu) ∼ -1.3, but z(CBF) ∼ -0.3 (similar discrepancies are observed for cerebellar white matter).

**Table 1:** Region clustering of energetics metrics for the older cohort in structural subdivisions of the brain.

**Structural subdivision**	**Rep**	**rAG**	**z(CMRglu)**	**z(CBF)**
**Basal ganglia**	Lower in caudate, globus pallidus, and SN; higher in anterior putamen	High	Generally above brain average	Generally below brain average
**Cerebellum**	Low	Low	Significantly below brain average	Below brain average
**Frontal lobe**	Generally high, especially in middle and inferior frontal gyrus	Generally below brain average	Generally above brain average	Above brain average
**Parietal lobe**	Below average in superior parietal lobule; brain average in postcentral gyrus; higher in inferior parietal lobule, precuneus, and supramarginal gyrus	Slightly low, except in precuneus (slightly high) and superior parietal lobule (brain average)	Slightly high, except in postcentral gyrus and superior parietal lobule (brain average)	Generally above brain average
**Occipital lobe**	Brain average	Slightly elevated	Slightly above brain average	Slightly below brain average
**Temporal lobe**	Limited clustering			
**Limbic lobe**	Low in entorhinal cortex, hippocampus, parahippocampal gyrus, and amygdala; slightly high in cingulate cortex	Brain average, except in rostral anterior cingulate cortex (below brain average)	Significantly below brain average, except in cingulate cortex (around brain average)	Below brain average, except in cingulate cortex (above brain average)
**Other regions brain regions (not shown in**Fig. 2**)**	Slightly low in insula; low in thalamus; very low in cerebral white matter	Slightly low in insula and WM; slightly high in thalamus	Brain average in thalamus; slightly below average in insula; significantly below average in cerebral white matter	Brain average in insula; below brain average in thalamus and cerebral white matter

### 3.2 Age-related trends in rEP and rAG

**Fig. 3:**
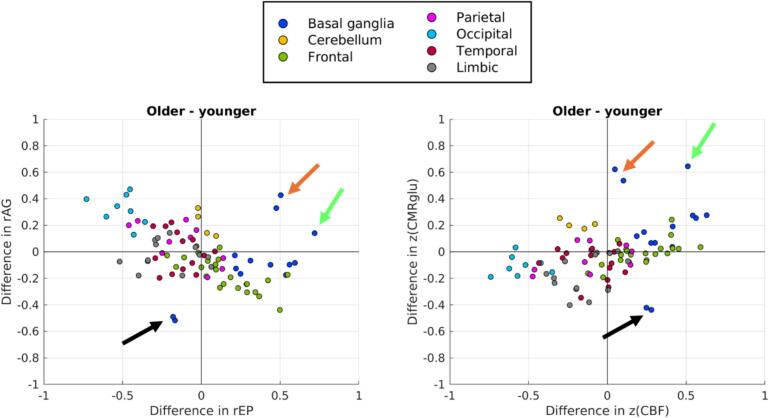
Directional shifts in the space of [rEP, rAG] (left) and [z(CMRglu), z(CBF)] (right). Arrows indicate regions of notable shift: bilateral caudate (black); bilateral SN (orange); left globus pallidus (green)—all parts of the basal ganglia group. (For interpretation of the references to colour in this figure legend, the reader is referred to the web version of this article.)

Fig. 3 showcases the shift of z(CMRglu), z(CBF), rEP, and rAG as a function of age. The most noticeable changes include bilateral caudate (black arrow), where rAG drops significantly with a smaller decrease in rEP; this stems from a large drop in z(CMRglu) combined with a smaller increase in z(CBF). In bilateral SN (orange arrow) and left globus pallidus (green arrow), both rEP and rAG demonstrate relatively large increases, mostly due to increases in z(CMRglu).

Other more subtle trends appear largely driven by changes in z(CBF), while z(CMRglu) remains relatively stable. Increases in z(CBF) thus lead to a decrease in rAG and small increase in rEP; this appears mostly confined to frontal regions, with the most noticeable changes in middle frontal gyrus. Conversely, decreases in z(CBF) lead to increases in rAG and decreases in rEP, which is primarily observed in occipital regions.

### 3.3 SSM-PCA age-related patterns in rEP and rAG

**Fig. 4:**
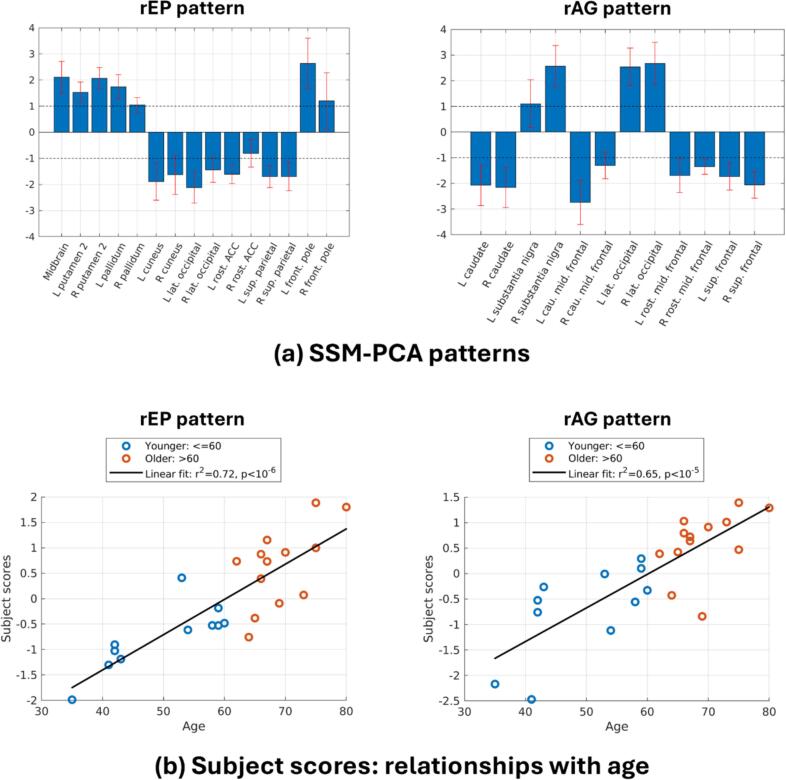
**(a)** SSM-PCA spatial covariance patterns for rEP and rAG that maximally separate younger vs. older subjects. Regions are only shown if their spatial weight, w, satisfies $\left|w\right|-\sigma \left(w\right)>1$, where σ(w) is the standard deviation of the weight, determined from five-fold cross-validation. For bilateral regions, both sides are shown if one side satisfies the condition above. **(b)** Relationships between the corresponding subject scores of each pattern (i.e., pattern expression) and age.

Combined spatial patterns are obtained each for rEP and rAG and pass cross-validation, with each pattern significantly separating younger vs. older subjects (rEP pattern: p = 0.0001, Cohen’s d = 1.88; rAG pattern: p = 0.0004, Cohen’s d = 1.66). Fig. 4a summarizes these spatial patterns and Fig. 4b plots the associated subject scores as a function of age, demonstrating strong age relationships with pattern expression strength (rEP pattern: r^2^ = 0.72, p < 10^-6^; rAG pattern: r^2^ = 0.65, p < 10^-5^). These patterns thus provide quantitative specificity to the qualitative age-related directional shifts observed in Fig. 3; the combination of regional changes in each pattern form spatial networks of energetic changes with age.

Based on these spatial patterns, rEP increases with age are most strongly observed in middle frontal gyrus, putamen, globus pallidus, and brainstem, whereas decreases are observed in lateral occipital cortex, cuneus, superior parietal lobule, and ACC. The strongest rAG increases with age are observed in SN, superior parietal lobule, and several occipital regions, whereas decreases are observed in caudate and frontal regions. In both patterns, the directionality of the regional weights exhibits bilateral symmetry. In general, the magnitudes are also similar for each side, though for the rEP pattern the magnitude of the weights tend to be slightly greater for the left side.

**Fig. 5:**
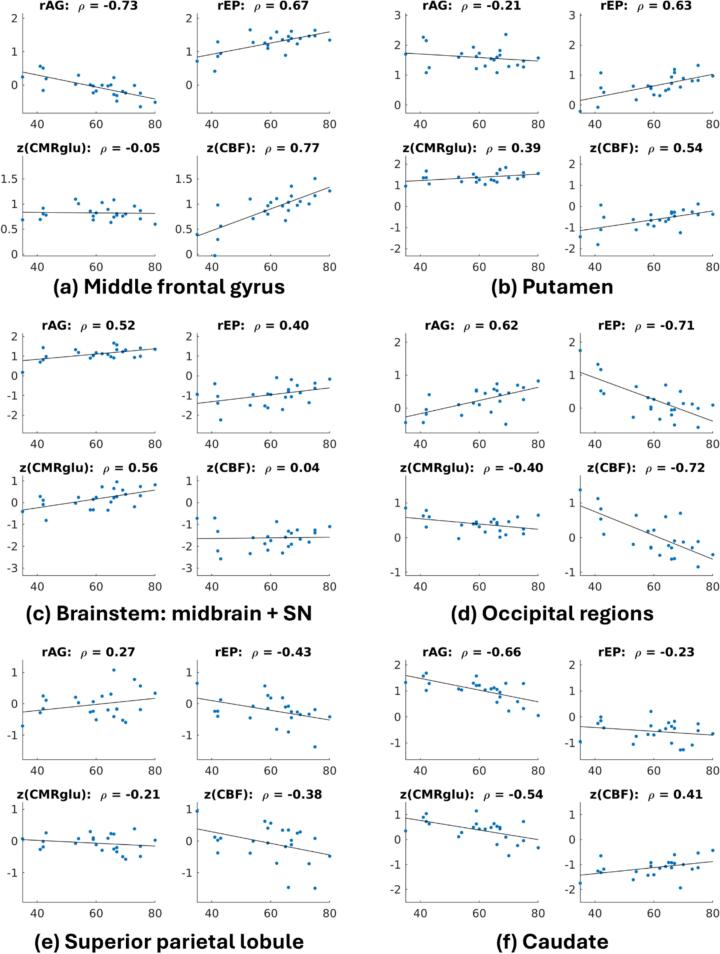
Relationships between brain energetics measures in age in representative regions. Linear regression fits and Pearson’s correlation coefficients are shown, but not p-values, as these relationships are presented for illustrative purposes and are not meant to be strict statistical tests for associations.

The linear combination of these changes thus strongly accounts for age-related variance in rEP and rAG. However, analyzing post hoc correlations of z(CMRglu) and z(CBF) with age within these individual regions provides physiological context for how these age-related changes manifest (Fig. 5). While these univariate correlations are not as statistically rigorous as the multivariate SSM-PCA analysis, they are nevertheless informative in decomposing the observed rEP and rAG changes into relative changes in CMRglu and/or CBF for representative regions, ultimately enhancing the interpretability of the underlying physiological changes. For instance, rEP increases in the middle frontal gyrus are mostly due to relative increases in CBF without much change in CMRglu, thus also leading to decreased rAG (Fig. 5a). rEP increases in the putamen stem from relative increases in both CBF and CMRglu, meaning rAG remains stable (Fig. 5b). Conversely, rEP increases in brain stem are mostly due to increases in CMRglu, leading to a corresponding increase in rAG (Fig. 5c). Decreasing rEP in occipital (Fig. 5d) and superior parietal regions (Fig. 5e) are mostly related to relative decreases in CBF, which is therefore accompanied by increasing rAG. Finally, in the caudate relative increases in CBF are coupled with relative decreases in CMRglu (Fig. 5f), which combine to yield a strong decrease in rAG.

A supplementary PLSC analysis which derives spatial patterns from the correlations between rEP and the clinical measures of age and sex identified a single significant pattern (Fig. S1) whose subject scores strongly relate to age (r^2^ = 0.77, p < 10^-7^) but not sex. The spatial pattern weights between the PLSC and SSM-PCA patterns strongly correlate (ρ = 0.84, p < 10^-26^). Applying PLSC to rAG (Fig. S2) yielded a similarly strong relationship with age (r^2^ = 0.68, p < 10^-6^) and high spatial similarly between PLSC and SSM-PCA patterns (ρ = 0.74, p < 10^-17^). This provides validation of the topological rEP and rAG aging profiles derived from SSM-PCA and offers further support that the expression of these profiles increases relatively linearly with chronological age.

### 3.4 Relationships to neurodegenerative disease-related patterns

Table 2 reports associations between the age-related rEP/rAG of this work and the previously derived PDRP, PDCP, and ADRP patterns. The regional weights in the rEP pattern showed a weak, but significant relationship with PDRP weights, remaining significant after Bonferroni correction (Spearman’s ρ = 0.28, adjusted p = 0.03), whereas the rAG pattern regional weights showed a similar correlation strength with PDCP regional weights (Spearman’s ρ = 0.27, adjusted p = 0.04). For ADRP, a weak association was observed with rAG pattern regional weights, but this relationship was no longer significant after multiple comparisons correction (uncorrected p = 0.03). The rEP–PDRP relationship mostly stems from shared positive weights in the putamen, globus pallidus, and SN and shared negative weights in superior parietal lobule, and for rAG–PDCP the relationship stems from common positive weightings in the cerebellum and SN and common negative regional weights in middle and superior frontal gyri. The modest spatial correlations observed thus arise from concordance between aging and disease profiles only in a subset of structural subdivisions, which is visualized in Fig. S3 of the Supplementary Information.

**Table 2:** Associations between age-related rEP/rAG patterns and neurodegenerative disease-related patterns.

	Spatial weights		Subject scores	
	Spearman’s ρ	Adjusted p	Pearson’s ρ	Adjusted p
rEP vs. PDRP	**0.28**	**0.03**	**0.55**	**0.03**
rEP vs. PDCP	0.18	0.37	0.43	0.20
rEP vs. ADRP	0.04	1.00	0.48	0.10
rAG vs. PDRP	0.09	0.95	0.31	0.60
rAG vs. PDCP	**0.27**	**0.04**	0.22	0.89
rAG vs. ADRP	0.25	0.18	0.46	0.14

**Fig. 6:**
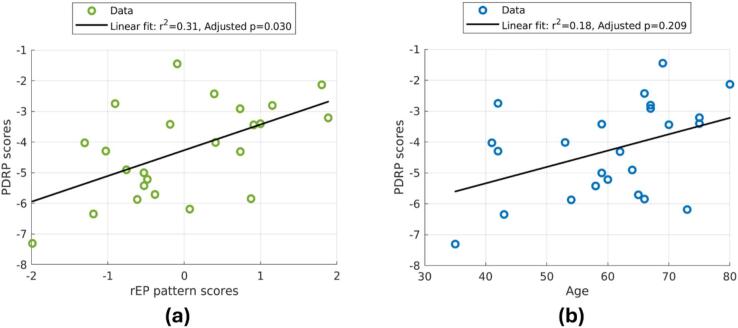
**(a)** The relationship between PDRP subject scores and rEP pattern subject scores in our healthy control cohort. **(b)** The relationship between PDRP subject scores and age.

In terms of subject scores, only rEP and PDRP demonstrated significantly covarying pattern expression after multiple comparisons correction (graphically illustrated in Fig. 6a). This association could exist because of a common relationship with age: Fig. 6b reveals that indeed PDRP subject scores also vary with age, although this relationship is weaker than the rEP–PDRP subject score relationship (r^2^ = 0.18 vs. r^2^ = 0.31). As a result, the expression of the age-related rEP pattern better explains the variance in the PDRP expression than chronological age itself.

## 4 Discussion

### 4.1 rEP and rAG as measures of brain energetics

By examining concurrence and discordance of CMRglu and CBF, we defined two orthogonal measures relevant to brain energetics: relative energy production (rEP) and relative aerobic glycolysis (rAG). These measures are sensitive to the *spatial distribution* of CMRglu and CBF across the brain rather than to absolute values in metabolism and blow flow. Therefore, changes in these measures signify a restructuring of energy production and/or associated energy production mechanisms, rather than tracking absolute increases or decreases.

Relevant to our analyses of aging, this implies that a change in rEP does not represent any global change in CMRglu and CBF with age. Instead, it addresses the question of how energy is distributed across the brain if one is limited to a given global amount of energy production. While it is becoming increasingly clear that metabolism decreases in the brain with age due to bioenergetic maladaptations on the cellular level (Yang et al., 2023, Na et al., 2025), rEP arguably provides a more nuanced, network-like description of how the aging brain restructures its energy delivery and utilization regionally across the brain. rAG instead focuses on the discordance between relative CMRglu and relative CBF. Again, the relative nature means that rAG compares the spatial distributions of CMRglu and CBF. Positive rAG means that relative CMRglu exceeds relative CBF—regardless of what the absolute levels are.

We have taken the interpretation that rAG > 0 is a marker of a higher degree of aerobic glycolysis, relative to the average level of aerobic glycolysis in the brain. Our analysis assumes that spatial variability in CMRO2 is exclusively provided by spatial variability in CBF, i.e. z(CBF) = z(CMRO2). Since CMRO2 is a function of CBF and oxygen extraction fraction (OEF), this assumption is dependent on OEF being uniform in the brain in healthy individuals, which is supported by the PET and MRI literature (Gauthier and Hoge, 2012, Wise et al., 2013, Cho et al., 2021, Fan et al., 2020). Despite this, a newer MRI-based method suggests a weak coupling between CBF and OEF with aging, meaning OEF may act in a neurovascular unit-based compensation for age-related changes in CBF (Wu et al., 2024). If true, this neutralizing effect of OEF would mean our observed changes z(CBF) would be greater than changes in z(CMRO2), thus affecting rAG interpretations. Specifically, this would manifest as follows: (i) a local increase in CBF partially offset by a local decrease in OEF results in z(CBF) > z(CMRO2), meaning rAG is negatively biased by a constant OEF assumption; (ii) a local decrease in CBF partially offset by a local increase in OEF results in z(CBF) < z(CMRO2), meaning rAG is positively biased by a constant OEF assumption. Furthermore, the quantitative OEF method used by (Wu et al., 2024) makes several assumptions—most notably a population-derived arterial hemoglobin concentration—that may not be valid with aging and thus would confound the OEF–CBF relationship. More importantly, this reported coupling was found to be weak; therefore, the majority of age-related variance in z(CBF)–z(CMRglu) discordance (i.e., rAG) we observe is indeed likely related to z(CMRO2)–z(CMRglu) discordance.

Additionally, rAG captures any discordance between z(CBF) and z(CMRglu). Our pattern analysis methodology requires changes in rAG to be consistently different between older and younger subjects and cross-validation iterations. Therefore, image noise and/or outlying subjects are unlikely to significantly impact the profile of age-related rAG changes. However, z(CBF)–z(CMRglu) discordance could arise from biological sources other than aerobic glycolysis. Most notably, glial and neuronal metabolism are indistinguishable in FDG-PET measurements, so changes in rAG may be due to a combination of changes in aerobic glycolysis and glial metabolism. This is considered in the biological interpretations of our findings in the sections that follow.

Finally, rEP and rAG are functions of z(CMRglu) and z(CBF), meaning age-related changes may be driven by z(CMRglu), z(CBF), or both. For instance, increasing rAG can result from any of the following, paired with a corresponding change in rEP: (i) both z(CMRglu) and z(CBF) increase, but z(CMRglu) increases more (increasing rEP); (ii) z(CMRglu) increases while z(CBF) is stable (increasing rEP); (iii) z(CMRglu) increases while z(CBF) reduces (approximately stable rEP); (iv) z(CMRglu) is stable while z(CBF) reduces (decreasing rEP); (v) both z(CMRglu) and z(CBF) decrease, but z(CBF) decreases more (decreasing rEP).

Scenarios (iii)–(v) are particularly relevant to the proposed link between mitochondrial dysfunction and aerobic glycolysis; lowered mitochondrial function would lead to reduced oxidative phosphorylation, meaning either glycolysis would increase to meet energy demands in scenario (iii), or energy production would reduce overall in scenarios (iv) and (v). In contrast, scenarios (i) and (ii) may indicate increased aerobic glycolysis independent of mitochondrial dysfunction, but equally could signal increased glucose metabolism by glial cells instead of increased neuronal glycolysis.

### 4.2 Regional variability of rEP and rAG in healthy individuals

rEP was consistently highest within regions of the default mode network (DMN), as was expected for the resting state. Similarly, lower rEP was observed in limbic regions, which may be expected in in the absence of emotional stimuli. Transverse temporal gyrus has the highest rEP of all regions; however, since this region forms a large portion of the auditory cortex this may not reflect resting energy levels, since these data were acquired under the high noise environment of a PET/MRI scanner.

rAG was highest in the basal ganglia, most notably in the putamen and SN. Elevated levels of absolute aerobic glycolysis (AG) may occur as a protective mechanism against oxidative stress in highly energetic neurons (Brand and Hermfisse 1997), in particular within the somata of neurons (Wei et al., 2023). The high density of synapses from dopaminergic neurons and large axonal field within the SN pars compacta (SNc) is hypothesized to place a large energetic demand on this region (Bolam and Pissadaki 2012). Additionally, mitochondrial density has been recently shown to be high in the putamen (Mosharov et al., 2025), which would lead to high levels of reactive oxygen species. Given the high number of projections from SNc to putamen, the observed high rAG in these regions may partly reflect a protective mechanism of aerobic glycolysis across this axis.

Low rAG regions included cerebellum, where CBF is below average but CMRglu is even further below average. Additionally, low rAG was found in several regions of the temporal lobe, specifically temporal pole and superior temporal gyrus, as well as in rostral ACC; in these cases, the lowered rAG is driven by a higher-than-average CBF that exceeds relative CMRglu.

Using a similarly conceived proxy for AG termed the glycolytic index (GI), (Vaishnavi et al., 2010) also found higher GI in striatum and lowered GI in cerebellum. However, high GI was also found within regions of the DMN and lateral temporal gyrus and low AG in inferior temporal gyrus, which our results did not support. However, their data were acquired on a much younger cohort (20–33 years, mean 25.4 ± 2.6); trends with age discussed below may partly explain these apparent discrepancies.

### 4.3 Aging effects on rEP and rAG

The visual analysis of Fig. 3 and SSM-PCA analysis of Fig. 4 demonstrated a widespread restructuring of rEP and rAG with age. The supplementary PLSC analyses (Figs. S1 and S2) demonstrate a high degree of spatial similarity to the SSM-PCA patterns, thus improving the statistical robustness of the rEP and rAG aging profiles identified. Among the most striking results include a large age-related decrease in rAG in frontal regions, from slightly above average rAG in subjects closer to age 40 and decreasing to well-below brain average closer to age 80 (Fig. 5a). Similarly, caudate rAG steeply declines from an elevated level to around brain average by age 80 (Fig. 5f). Therefore, the results of Vaishnavi et al. (2010) having elevated frontal lobe and caudate AG in subjects closer to age 25 are consistent with this age-related decrease. Additionally, assuming a global reduction in absolute AG with age based on the results of (Goyal et al., 2017), the observed decrease in caudate/frontal rAG would indicate these regions decreases even faster than the whole-brain average—also supported by their regional investigations.

By contrast, age-related increases in rAG appeared in occipitoparietal regions. Lateral occipital cortex, lingual gyrus, and pericalcarine cortex (Fig. 5d) and superior parietal lobule (Fig. 5e) in particular demonstrated rAG increases and rEP decreases; we hypothesize these trends are suggestive of mitochondrial dysfunction, as z(CMRglu) levels are slightly declining with age while z(CBF) experiences a more substantial decrease. However, more direct measures of in vivo mitochondrial function are required to substantiate these claims. These occipital regions largely overlap with the visual cortex, whereas superior parietal lobule is more associated with sensorimotor integration (Wolpert et al., 1998). The rEP and rAG findings in these regions may provide a neurophysiological contribution to the well-known age-related impairments in sensorimotor performance with aging, which may be related and/or interacting with other age-related changes to sensorimotor integration uncovered from magnetoencephalography (Piitulainen et al., 2018), transmagnetic stimulation (Degardin et al., 2011, Brown et al., 2018), and fMRI (Yoshimura et al., 2020).

SN and midbrain also displayed an increase in both rEP and rAG with age, exclusively driven by increasing z(CMRglu) (Fig. 5c). This increase does not fit with the theory of mitochondrial dysfunction, but because CMRglu reflects metabolism in both neurons and glial cells this may indicate an increase in glial activity with age, supported by mice models and human ex vivo data in healthy individuals (Jyothi et al., 2015, Moca et al., 2022).

Finally, while auditory regions—especially bilateral transverse temporal gyri—are not amongst the highest weighted regions in the rEP and rAG patterns of Fig. 4a, their weightings are non-negligible (60th percentile for the rEP pattern and 75th percentile for rAG). Since the high noise scanning environment evidently activates these regions, age-related hearing loss would introduce a potential confound through differential perception of scanner noise. However, the age relationships of these patterns are inferred from the linear combination of rEP/rAG changes, so the moderate weighting in the auditory cortex only has a small effect on the overall age relationship. In fact, repeating our analysis with bilateral transverse temporal gyri removed only slightly weakened the age relationship of the identified patterns: for rEP we found r^2^ = 0.71, p < 10^-6^ compared to r^2^ = 0.72, p < 10^-6^ in the original pattern; for rAG we found r^2^ = 0.62, p < 10^-5^ compared to r^2^ = 0.65, p < 10^-5^ in the original pattern.

### 4.4 Potential brain energetic relationships between healthy aging and neurodegeneration

Correlational analysis of topological similarities between age-related rEP/rAG patterns of this work and previous disease-related patterns derived from CMRglu data reveal that the strongest association exists between rEP spatial weights and those of the PDRP (Table 2); particularly shared positive weights in the basal ganglia along with shared negative weights in superior parietal lobule (c.f. Figs. S1 and S2 of the Supplementary Information). Since rEP and CMRglu are highly correlated and thus are both sensitive to changes in energy production, topological similarities in these regions suggest some degree of overlap between the regional energetic changes involved in healthy aging and pathological changes involved in PD. However, since the spatial overlap is localized to select regions, this means aging effects are also *not* present in a large subset of regions implicated in PD pathology, and the spatial correlation reported is thus modest in magnitude (ρ = 0.28).

The PDRP is able to discriminate PD vs. healthy controls via subject scores by capturing metabolic differences between the healthy and diseased brain, but its subject scores also tend to correlate with disease duration, motor disease severity, and the effects of symptomatic therapy (medication and surgery) within PD subjects (Ma et al., 2007, Tomše et al., 2017)—meaning it is also sensitive to progression and expression of disease. Likewise, within healthy individuals the PDRP expression has considerable variation and tends to overlap with expression in those with PD (i.e., the lowest-expressing PD subjects have lower subject scores than the highest-expressing HC subjects). This may suggest a continuum in the metabolic topology between the healthy and diseased brain.

As age is the highest risk factor for PD, it is not surprising that we observe a correlation between PDRP expression and age in our cohort (Fig. 6b), although all subjects have a negative subject score (as might be expected for a healthy cohort). However, Fig. 6a reveals that the expression of age-related rEP changes explains more variance in PDRP expression than chronological age alone. Stated differently, if one takes PDRP expression in healthy controls as a proxy for metabolic similarity to a brain with PD, this means that subjects who have a higher expression of age-related rEP changes are metabolically more similar to the PD subjects, despite not having the disease. These associations suggest a possibility that a subset of the rEP changes involved in aging—especially changes in regions where PD metabolic pathology occurs—contribute to a propensity for future pathology. However, a longitudinal analysis would be required to investigate these claims.

Conversely, the age-related rAG pattern shows no relationship to the PDRP, indicating the topographic profile of age-related rAG changes is distinct from the set of metabolic alterations associated with motor dysfunction in PD. In fact, rAG increases with age in occipital regions, which is opposite to CMRglu decreases observed in PD, and rAG decreases in frontal regions, which are not prominently featured in the PDRP. Instead, a significant association was found between the age-related rAG pattern and the PDCP, a secondary PD-related metabolic pattern that tracks with cognitive decline in PD (Huang et al., 2007), primarily due to common positive weightings in the cerebellum and SN and negative weightings in the frontal cortex. However, the subject scores of these patterns do not covary. This implies that the expression of age-related rAG changes does not explain the variance in PDCP expression in healthy controls. Additionally, PDCP expression was also not found to correlate with age (results not shown). Furthermore, the PDCP is derived from CMRglu data, so overlap of spatial weights in the rAG pattern may instead signal energy production mechanisms in healthy aging that could contribute to future metabolic abnormalities that present in cognitive impairment in PD. For instance, since AG provides a protective mechanism from oxidative stress (Brand and Hermfisse, 1997, Wei et al., 2023), the reduction in rAG in the age-related pattern could lead to an accumulation of reactive oxygen species with aging that, in turn, may contribute to the neuronal hypometabolism observed in the PDCP. This overall weaker finding with PDCP compared to PDRP also may be attributed to PDCP expression appearing later in the disease process (Huang et al., 2007).

Finally, no significant relationships are observed between the age-related patterns and the ADRP. This may be because the onset of AD is on average later than PD (Hou et al., 2019), and/or the cumulative effect of age-related energetics changes may not strongly relate to downstream metabolic alterations in AD. While a previous study demonstrated a spatial correlation between the distributions of aerobic glycolysis in young adults and amyloid beta deposition in AD patients (Vlassenko et al., 2010), our rAG spatial pattern instead captures the *changes* in AG with age rather than the distribution of AG, and the ADRP is different from distributions of amyloid beta deposition, so we did not expect to reproduce this particular result.

### 4.5 Limitations

Despite a relatively wide age range (35–80 years), our dataset includes a relatively low number of subjects. A small dataset increases the likelihood of overfitting when searching for age-related changes; we have attempted to minimize this effect through (i) combining robust image denoising (Cheng et al., 2022) and utilizing regional data to maximize the signal-to-noise ratio of our dataset, (ii) using five-fold cross-validation when deriving our SSM-PCA patterns, and (iii) validating these patterns via a supplementary PLSC analysis, which yielded highly correlated spatial patterns. External validation of these patterns using a separate dataset of aging healthy controls should be a target for future work. Furthermore, several disease-related SSM-PCA metabolic patterns have been identified and validated with comparable subject numbers, including the PDCP (Huang et al., 2007: 15 PD subjects), ADRP (Teune et al., 2014: 15 diseased subjects and 18 healthy controls), the dementia with Lewy Bodies related pattern (Perovnik et al., 2022: 20 diseased subjects and 20 healthy controls), and the behavioral variant of frontotemporal dementia pattern (Nazem et al., 2018: 10 diseased subjects and 10 healthy controls).

Our results are also limited by a cross-sectional study design. The changes in rEP and rAG identified were found to associate with age; longitudinal follow-up with subjects would be required to causally link these changes with age. The comparison between age- and disease-related covariance patterns would also benefit from a longitudinal study design: our hypothesis of age-related brain energetics changes in select regions translating to an increased propensity for pathology was based on spatial correlation between age- and disease-related patterns, so observing if the subject-specific expression of these patterns increases in concert in follow-up and/or if pattern expression predicts future conversion to disease would lend credence to our hypothesis.

The pulse sequence used to obtain CBF images had a fixed post-label delay (PLD) of 1525 ms for all subjects, despite recommendations for a longer PLD in elderly subjects (Hu et al., 2020). This choice stemmed from matching the PLD to another ASL sequence in the scanning session (not relevant to this study), which, due to technical limitations, could not have a longer PLD. This likely has an effect on the quantitative accuracy of absolute CBF values. However, our rEP and rAG measures are derived from relative CBF, which only requires accuracy of the spatial distribution of CBF. A more recent multimodal PET/MRI study in elderly subjects also found that a single 1800 ms PLD provided the strongest correlation between regional ASL-derived CBF and that from the gold standard of [^15^O]water PET imaging, and that correlation strength generally decreased for higher PLDs (Fu et al., 2026). This suggests the lower-than-optimal PLD for elderly subjects used in our study likely captures the correct spatial distribution of CBF despite the potential for absolute quantification errors.

Additionally, rEP and rAG should be taken as proxy measures of energetics as they are derived from neuroimaging data which are integrated over the metabolic and hemodynamic processes of millions of neurons. Our observations thus serve to provide a coarse-grained understanding of directional shifts in energetics within aging. As such, we can only provide in vivo evidence that may lend support to existing and future in vitro, preclinical, and ex vivo studies, which all offer greater specificity towards the cellular processes that may underlie these changes. Acquiring rEP and rAG data alongside Magnetic Resonance Spectroscopy (MRS) data would be particularly valuable for validating the specificity of rEP and rAG towards energy production and aerobic glycolysis, respectively.

Relatedly, as with any analysis involving FDG, quantitative values aggregate metabolism across neurons and glial cells. This means one cannot definitively identify the mechanism(s) responsible for the changes in rEP and rAG, especially when these changes are related to changes in CMRglu. In a study of aging in mice, age-related changes in FDG uptake were found to be more closely related to changes in glial rather than neuronal metabolism—especially later in the aging process (Bartos et al., 2024)—but it is unknown whether these changes translate to humans. The interpretations provided in our discussion above thus are necessarily speculative, but we have attempted to correlate them with external support, such as results from other imaging modalities or ex vivo data.

## CRediT authorship contribution statement

**Connor W. J. Bevington:** . **Sahib Dhaliwal:** Investigation, Data curation. **Jessamyn McKenzie:** Investigation, Data curation. **A. Jon Stoessl:** Writing – review & editing, Methodology, Conceptualization. **Vesna Sossi:** Writing – review & editing, Supervision, Methodology, Conceptualization.

## Funding

This work was supported by Parkinson Canada [grant number GR032198, 2024]; Pacific Parkinson’s Research Institute [grant number GR014738, 2018]; the Natural Science and Engineering Research Council [grant number GR010121, 2017].

## Declaration of competing interest

The authors declare that they have no known competing financial interests or personal relationships that could have appeared to influence the work reported in this paper.

## Data Availability

Data will be made available on request.

## Footnotes

1 By dint of the z-score normalization in equation (2), rEP and rAG are already zero-centred across regions.
