# Supplementary material for: Age-related changes to brain energetics revealed by PET/MRI imaging
Source: Neuroimage Clin. 2026 May 27;50:104014. doi: 10.1016/j.nicl.2026.104014 (PMC13241851; doi:10.1016/j.nicl.2026.104014)
Supplement: Supplementary Data 1 [file mmc1.docx]

**Supplementary Information for “Age-related changes to brain energetics revealed by PET/MRI imaging”**

**Partial Least Squares Correlation analysis (PLSC) applied to rEP and rAG**

PLSC was performed as described in Krishnan et al. (2011) separately for rEP and rAG. Briefly, this implementation of PLSC computes the correlation matrix between normalized imaging data contained in $\boldsymbol{X}$ (subjects-by-regions) and normalized clinical data contained in $\boldsymbol{Y}$ (subjects-by-variables):

$\boldsymbol{R}\boldsymbol{=}\boldsymbol{Y}^{\boldsymbol{T}}\boldsymbol{X}$**.** (S1)

$\boldsymbol{R}$ thus has the dimensionality of variables-by-regions. In our case, we used age and sex as clinical variables. Singular Value Decomposition (SVD) is then applied to $\boldsymbol{R}$ to yield imaging (spatial) patterns in the columns of $\boldsymbol{V}$ and clinical patterns in the columns of $\boldsymbol{U}$:

$\boldsymbol{R=U}\boldsymbol{\Sigma}\boldsymbol{V}^{\boldsymbol{T}}$**.** (S2)

Spatial pattern and clinical pattern subject scores are obtained as usual:

$\boldsymbol{S}_{\boldsymbol{X}}\boldsymbol{=XV}$ (S3)

$\boldsymbol{S}_{\boldsymbol{Y}}\boldsymbol{=YU}$**.** (S4)

PLSC is optimized such that the first set of subject scores (i.e., the first column of $\boldsymbol{S}_{\boldsymbol{X}}$ and $\boldsymbol{S}_{\boldsymbol{Y}}$) maximally covary, meaning subjects who have a high expression of the spatial pattern also have a high expression of the clinical pattern. The remaining covariance is maximized for the second set of subject scores, and so on. Additionally, the clinical weights in each column of $\boldsymbol{U}$ are strongly related to the correlation between the spatial pattern subject scores and the corresponding clinical variables, therefore providing clinical context for the spatial pattern.

Random permutation is employed to assess the generalizability of the pattern sets. This is achieved by randomly shuffling the rows of $\boldsymbol{X}$ (i.e., subjects) while leaving $\boldsymbol{Y}$ unchanged. Applying equations (S1) and (S2) using this permuted $\boldsymbol{X}$ yields a set of null singular values in $\boldsymbol{\Sigma}$**.** Ten thousand permutations were run, and a pattern pair is deemed significant if its corresponding singular value exceeds the null singular values at least 95% of the time (i.e., p<0.05).

A separate bootstrap analysis is run to assess the stability of the regional weights. Here, the rows of $\boldsymbol{X}$ and $\boldsymbol{Y}$ are identically resampled with replacement before applying equations (S1) and (S2). This is repeated 10,000 times to build a distribution of regional weights in $\boldsymbol{V}$ from which the standard error is computed. A stability measure is defined as the original regional weight divided by its standard error. A stability measure greater than two suggests the corresponding region has a salient contribution to the overall pattern.

The results from applying PLSC to rEP and comparisons to the SSM-PCA pattern of the main text are presented in Fig. S1. A single significant pattern set is identified (p=0.0002) that explains 70.0% of the total covariance between the imaging and clinical measures. The clinical pattern has high weighting for age and minimal weighting for sex, meaning the spatial pattern weights reflect an age-related pattern. This also implies the variability in rEP is significantly better explained by age than sex. In line with these observations, the spatial pattern subject scores show a strong association with age (r^2^=0.77, p<10^-7^) and no significant relationship with sex. Furthermore, the spatial weights strongly correlate with those of the age-related SSM-PCA rEP pattern of the main text (ρ=0.84, p<10^-26^).


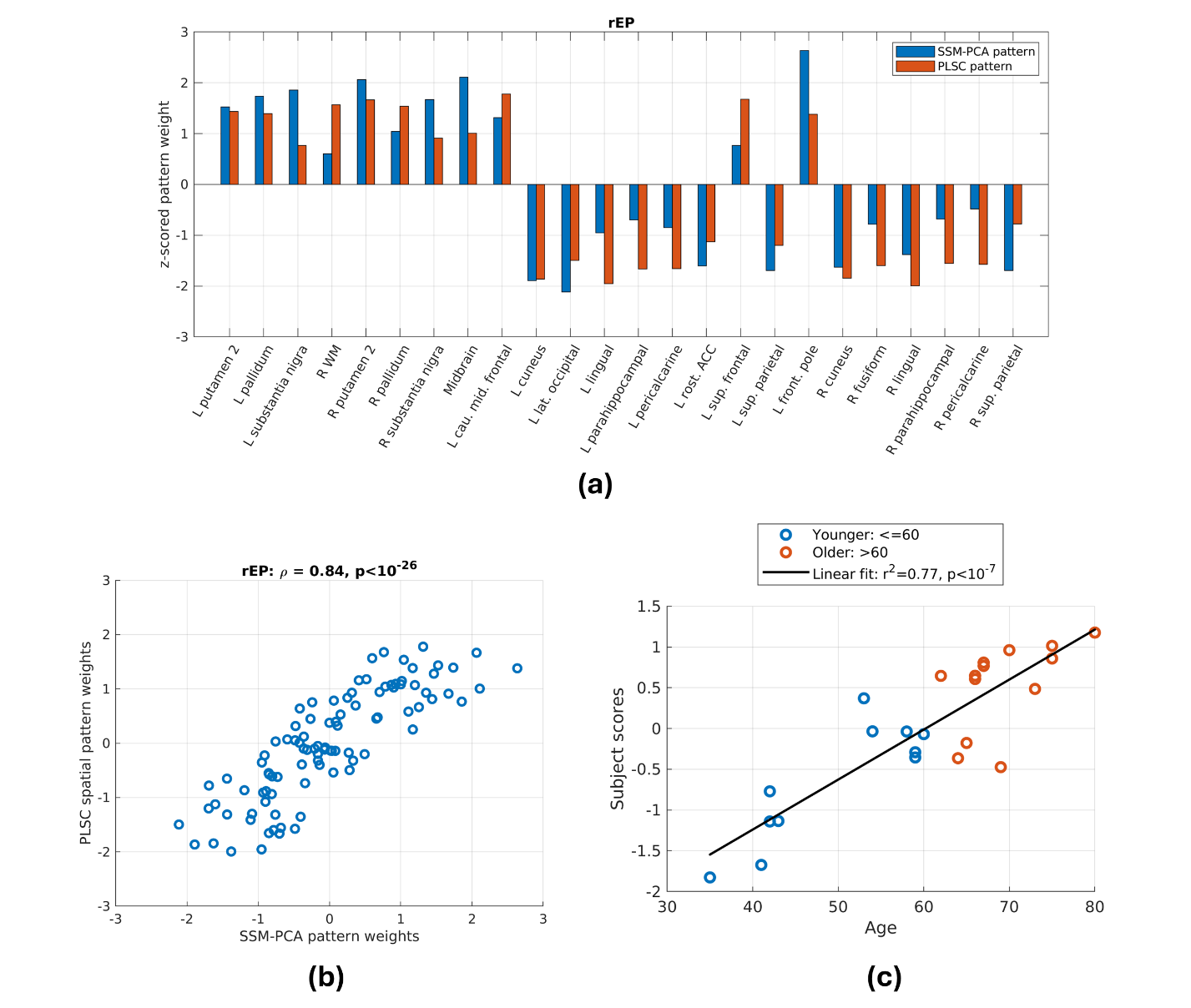


**Fig S1.** **(a)** Comparing the z-scored spatial weights between the age-related rEP patterns derived from SSM-PCA and PLSC. Only regions having a z-score > 1.5 for one of the patterns are shown for brevity; if the PLSC z-scored weight is > 1.5, it is only shown if it has a stability measure > 2. **(b)** Spatial correlation between the age-related rEP patterns derived from SSM-PCA and PLSC. **(c)** The relationship between the PLSC rEP pattern subject scores and age.

Similar results are observed for rAG, as shown in Fig. S2. A single significant pattern set is identified (p=0.0016) that explains 67.3% of the total covariance between the imaging and clinical measures. Clinical weightings are only high for age, again yielding a strong association with age (r^2^=0.68, p<10^-6^) and no significant relationship with sex. Finally, the spatial weights strongly correlate with those of the age-related SSM-PCA rAG pattern of the main text (ρ=0.74, p<10^-17^).


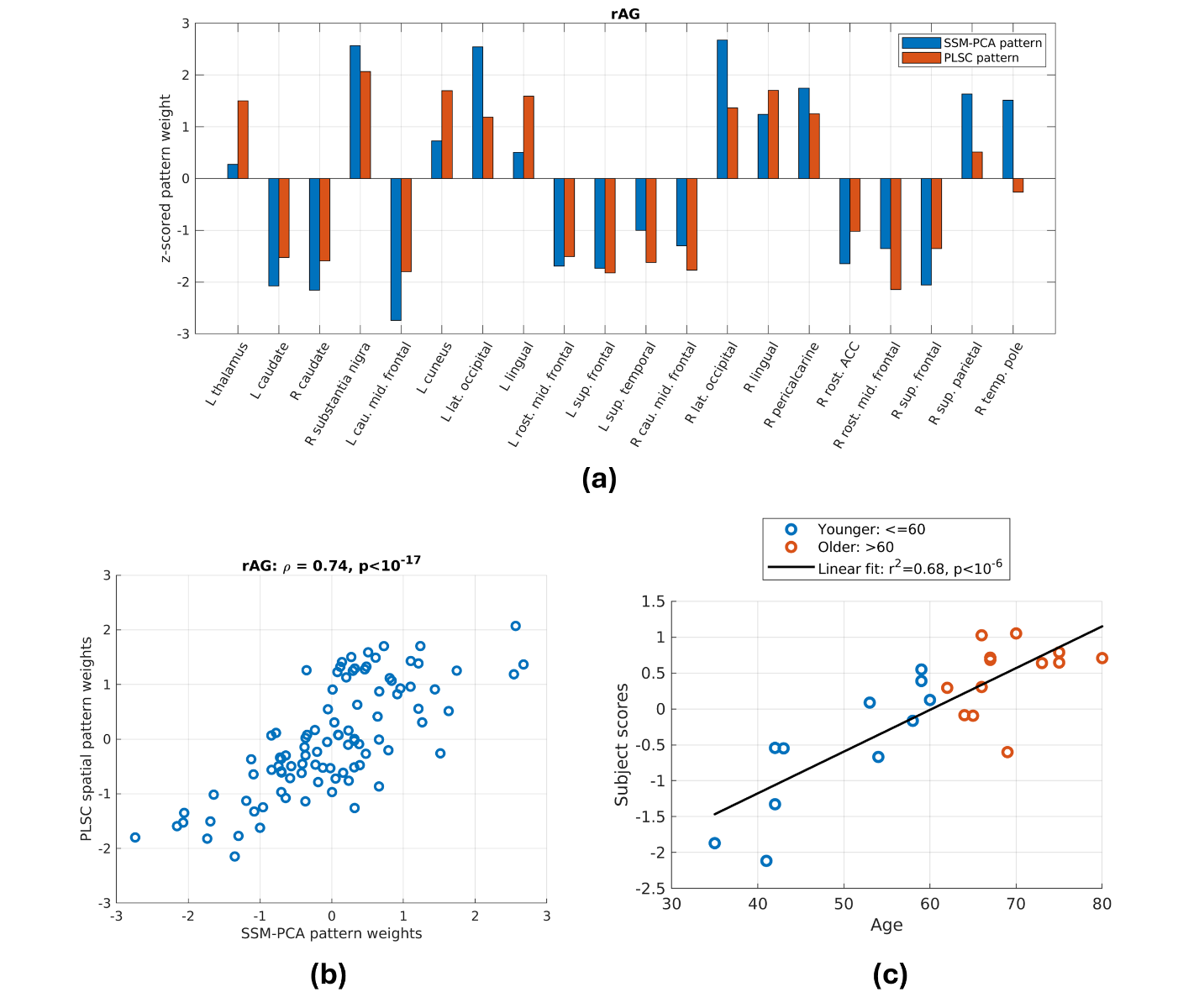


**Fig S2.** **(a)** Comparing the z-scored spatial weights between the age-related rAG patterns derived from SSM-PCA and PLSC. Only regions having a z-score > 1.5 for one of the patterns are shown for brevity; if the PLSC z-scored weight is > 1.5, it is only shown if it has a stability measure > 2. **(b)** Spatial correlation between the age-related rAG patterns derived from SSM-PCA and PLSC. **(c)** The relationship between the PLSC rAG pattern subject scores and age.

**Visualizing significant spatial correlations between age- and disease-related patterns**


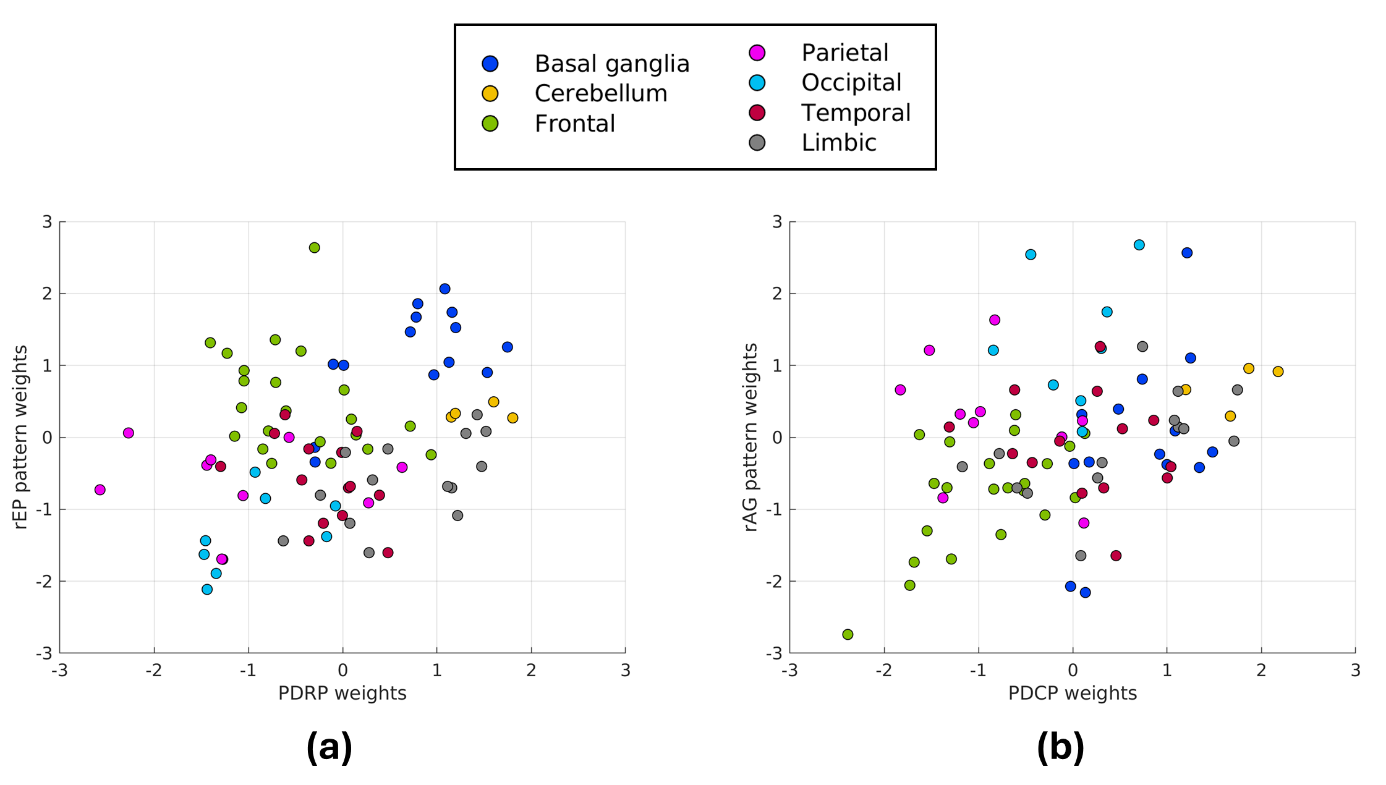


**Fig S3.** Correlation plots of the regional weights between **(a)** the PDRP and the rEP age-related pattern of this work, and **(b)** the PDCP and the rAG age-related pattern of this work. Weights have been z-scored and are colour-coded based on structural subdivisions of the brain.

**Table of ROIs used for regional analysis**

All ROIs are taken from Freesurfer segmentations of each subject’s T1 image in their native space, unless otherwise specified.

| **Name** | **Description** | **Notes** |
| --- | --- | --- |
| L WM | Left cerebral white matter |  |
| L cer WM | Left cerebellar white matter |  |
| L cer GM | Left cerebellar grey matter |  |
| L thalamus | Left thalamus |  |
| L caudate | Left caudate |  |
| L putamen 1 | Left anterior putamen | Subsegmented from the Freesurfer putamen ROI along the anterior–posterior axis |
| L putamen 2 | Left middle putamen |  |
| L putamen 3 | Left posterior putamen |  |
| L pallidum | Left globus pallidus |  |
| L hippocampus | Left hippocampus |  |
| L amygdala | Left amygdala |  |
| L vent. striatum | Left ventral striatum |  |
| L substantia nigra | Left substantia nigra | Hand-drawn |
| R WM | Right cerebral white matter |  |
| R cer WM | Right cerebellar white matter |  |
| R cer GM | Right cerebellar grey matter |  |
| R thalamus | Right thalamus |  |
| R caudate | Right caudate |  |
| R putamen 1 | Right anterior putamen | Subsegmented from the Freesurfer putamen ROI along the anterior–posterior axis |
| R putamen 2 | Right middle putamen |  |
| R putamen 3 | Right posterior putamen |  |
| R pallidum | Right globus pallidus |  |
| R hippocampus | Right hippocampus |  |
| R amygdala | Right amygdala |  |
| R vent. striatum | Right ventral striatum |  |
| R substantia nigra | Right substantia nigra | Hand-drawn |
| Midbrain | Midbrain | Hand-drawn |
| Pons | Pons | Hand-drawn |
| Medulla | Medulla | Hand-drawn |
| L bankssts | Left banks of the superior temporal sulcus |  |
| L cau. ACC | Left caudal anterior cingulate cortex |  |
| L cau. mid. frontal | Left caudal middle frontal gyrus |  |
| L cuneus | Left cuneus |  |
| L entorhinal | Left entorhinal cortex |  |
| L fusiform | Left fusiform gyrus |  |
| L inf. parietal | Left inferior parietal cortex |  |
| L inf. temporal | Left inferior temporal cortex |  |
| L isthmus cingulate | Left isthmus cingulate cortex |  |
| L lat. occipital | Left lateral occipital cortex |  |
| L lat. orbitofrontal | Left lateral orbitofrontal cortex |  |
| L lingual | Left lingual gyrus |  |
| L med. orbitofrontal | Left medial orbitofrontal cortex |  |
| L mid. temporal | Left middle temporal gyrus |  |
| L parahippocampal | Left parahippocampal gyrus |  |
| L paracentral | Left paracentral lobule |  |
| L pars opercularis | Left pars opercularis |  |
| L pars orbitalis | Left pars orbitalis |  |
| L pars triangularis | Left pars triangularis |  |
| L pericalcarine | Left pericalcarine gyrus |  |
| L postcentral | Left postcentral gyrus |  |
| L PCC | Left posterior cingulate cortex |  |
| L precentral | Left precentral gyrus |  |
| L precuneus | Left precuneus |  |
| L rost. ACC | Left rostral anterior cingulate cortex |  |
| L rost. mid. frontal | Left rostral middle frontal gyrus |  |
| L sup. frontal | Left superior frontal gyrus |  |
| L sup. parietal | Left superior parietal lobule |  |
| L sup. temporal | Left superior temporal gyrus |  |
| L supramarginal | Left supramarginal gyrus |  |
| L front. pole | Left frontal pole |  |
| L temp. pole | Left temporal pole |  |
| L trans. temporal | Left transverse temporal gyrus |  |
| L insula | Left insular cortex |  |
| R bankssts | Right banks of the superior temporal sulcus |  |
| R cau. ACC | Right caudal anterior cingulate cortex |  |
| R cau. mid. frontal | Right caudal middle frontal gyrus |  |
| R cuneus | Right cuneus |  |
| R entorhinal | Right entorhinal cortex |  |
| R fusiform | Right fusiform gyrus |  |
| R inf. parietal | Right inferior parietal cortex |  |
| R inf. temporal | Right inferior temporal cortex |  |
| R isthmus cingulate | Right isthmus cingulate cortex |  |
| R lat. occipital | Right lateral occipital cortex |  |
| R lat. orbitofrontal | Right lateral orbitofrontal cortex |  |
| R lingual | Right lingual gyrus |  |
| R med. orbitofrontal | Right medial orbitofrontal cortex |  |
| R mid. temporal | Right middle temporal gyrus |  |
| R parahippocampal | Right parahippocampal gyrus |  |
| R paracentral | Right paracentral lobule |  |
| R pars opercularis | Right pars opercularis |  |
| R pars orbitalis | Right pars orbitalis |  |
| R pars triangularis | Right pars triangularis |  |
| R pericalcarine | Right pericalcarine gyrus |  |
| R postcentral | Right postcentral gyrus |  |
| R PCC | Right posterior cingulate cortex |  |
| R precentral | Right precentral gyrus |  |
| R precuneus | Right precuneus |  |
| R rost. ACC | Right rostral anterior cingulate cortex |  |
| R rost. mid. frontal | Right rostral middle frontal gyrus |  |
| R sup. frontal | Right superior frontal gyrus |  |
| R sup. parietal | Right superior parietal lobule |  |
| R sup. temporal | Right superior temporal gyrus |  |
| R supramarginal | Right supramarginal gyrus |  |
| R front. pole | Right frontal pole |  |
| R temp. pole | Right temporal pole |  |
| R trans. temporal | Right transverse temporal gyrus |  |
| R insula | Right insular cortex |  |

**References**

Krishnan A, Williams LJ, McIntosh AR, Abdi H. Partial Least Squares (PLS) methods for neuroimaging: a tutorial and review. Neuroimage. 2011 May 15;56(2):455-75.
